# Single-mode lasers before the merger

**DOI:** 10.1038/s41377-026-02467-3

**Published:** 2026-09-08

**Authors:** G. Zito

**Affiliations:** Institute of Applied Sciences and Intelligent Systems, National Research Council of Italy, 80131 Napoli, Italy

**Keywords:** Semiconductor lasers, Photonic crystals

## Abstract

Merging bound states in the continuum offers a powerful route to compact lasers, but maximum Q is not always optimal. Peng and colleagues show that pre-merging conditions provide stronger threshold discrimination between target and competing modes, establishing modal selectivity as a key design rule for robust single-mode microscale BIC lasers.

A central problem in semiconductor lasers is deceptively simple: how can one increase optical performance without allowing unwanted modes to oscillate? In broad-area semiconductor lasers, increasing the emission area increases the available power, but it also favors many-mode oscillation and degraded beam quality. Photonic-crystal surface-emitting lasers have addressed this problem by engineering optical coupling inside a two-dimensional photonic lattice. In the large-area limit, recent works have shown that single-mode operation can be preserved by controlling Hermitian and non-Hermitian couplings so that higher-order modes experience larger radiation losses than the fundamental mode^1^. The lesson is broader than the particular device: a good laser is not defined only by the quality factor (Q) of one mode, but by the threshold margin separating that mode from all its competitors.

Recently published in *Light: Science & Applications*, the work of Peng and colleagues, “Robust single-mode laser via merging bound state in the continuum”, brings this principle into the realm of compact bound-state-in-the-continuum lasers^2^. Their device is a photonic-crystal slab laser based on InGaAsP multiple quantum wells. By tuning the air-hole diameter, the authors move accidental BICs in momentum space and merge them with a symmetry-protected BIC at the Γ point (Fig. 1). In an infinite photonic crystal, this merging broadens the high-Q region around Γ: instead of a narrow isolated high-Q point, the radiative loss remains suppressed over a larger domain of in-plane wave vector. This idea follows the topological picture of BICs as polarization singularities carrying conserved charges^3^ and the first experimental demonstration that merging multiple BICs can create ultrahigh-Q guided resonances robust to out-of-plane scattering^4^.

**Fig. 1:**
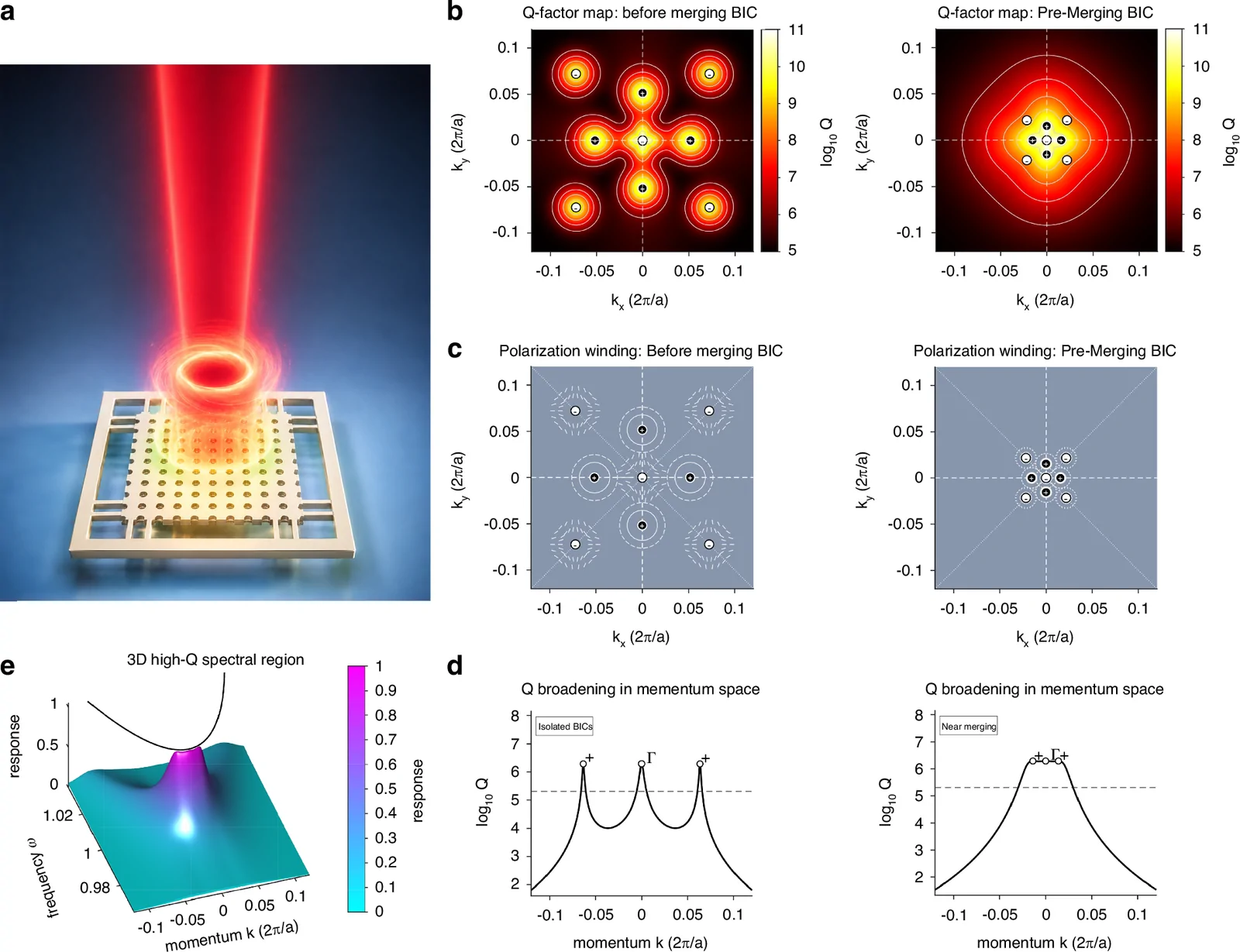
**Merging-BIC design for robust single-mode lasing**. **a** Artistic rendering of a miniaturized photonic-crystal slab laser, where a vortex-shaped lasing emission emerges from the active perforated membrane. **b** Calculated radiative quality-factor maps showing that BIC merging expands the high-Q region around the Γ point, providing a momentum-space route to enhance radiative protection and control modal competition in compact BIC lasers. **c** Evolution of the polarization/topological-charge configuration in momentum space before, and at BIC pre-merging. **d** Schematic evolution of the radiative quality factor in momentum space for isolated accidental BICs and for the near-merging regime, where the high-Q region broadens around the Γ point. The dashed line indicates a representative high-Q threshold. **e** Three-dimensional representation of the broadened spectral response associated with the high-Q region at normalized frequency

At first sight, one might expect the exact merging point to be the optimum laser design, because it provides the broadest radiative protection. Peng et al. show that this intuition is incomplete. In a finite photonic crystal, the continuous BIC band of the ideal infinite slab is quantized into discrete cavity-like modes. The desired fundamental state and nearby higher-order states are therefore not independent of the BIC landscape; they both sample it. If the high-Q region becomes too broad, it can protect the competing modes as well as the target mode (Fig. 2). The result is a lower threshold, but a weaker single-mode hierarchy.

**Fig. 2:**
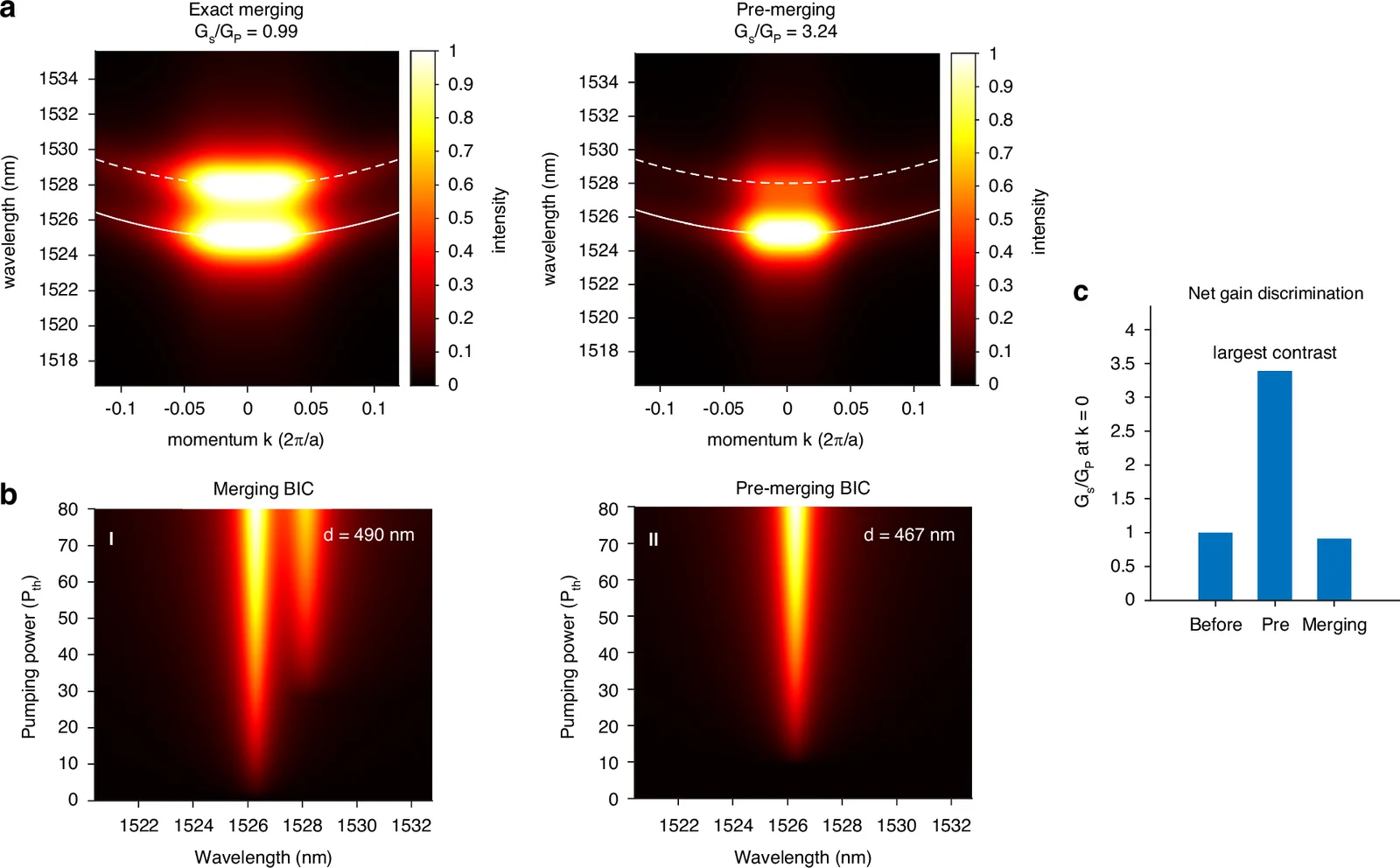
**From Q-factor maximization to modal discrimination in finite merging-BIC lasers**. **a** Phenomenological calculated two-mode gain maps illustrating how the pre-merging condition maximizes the contrast between the target s-like BIC-derived mode and a competing p-like mode. **b** Calculated lasing spectra as a function of pump power reproducing the contrast discrimination-concept on mode lasing: at merging BIC both s- and p-like modes have sufficient gain to lase (I), whereas the system transitions to robust single-mode emission up to 80 times threshold as the contrast between the modal gain increases at the pre-merging condition (II). **c** Corresponding modal gain discrimination, showing that the optimum laser is not necessarily obtained at the highest absolute Q, but where the target mode has the largest threshold advantage over competing channels

This is the key message of the paper: the most robust single-mode laser is obtained not exactly at the merging condition, but slightly before it. In this pre-merging regime, the fundamental s-like BIC-derived mode is already strongly protected, while the nearest p-like competing mode still experiences substantially larger loss. Peng et al. calculate that, in the pre-merging device, the threshold gain of the p-like competitor is more than three times larger than that of the s-like lasing mode. At exact merging, this contrast collapses to only about 1.3. Experimentally, this difference is decisive: the pre-merging device remains strictly single-mode up to 80 times threshold, whereas at the merging condition a higher-order BIC-derived mode begins to lase at high pump power.

This reframes the role of BIC merging in active devices. Merging BICs were originally attractive because they suppress radiative leakage and make high-Q resonances more robust against disorder and finite angular spread. Super-BIC lasers have already used this principle to reduce threshold by merging symmetry-protected and accidental BICs in momentum space^5^. Peng et al. add an active-laser criterion: one should not maximize Q blindly, but maximize the useful threshold contrast between the desired mode and the nearest competing modes. The relevant figure of merit is therefore not simply Q, but something closer to ${G}_{{\rm{s}}}/{G}_{{\rm{p}}}$ (Fig. 2), or more generally the separation between the first and second lasing thresholds under realistic gain saturation.

This distinction is especially important for miniaturized BIC lasers. Ideal BICs are Bloch modes of extended periodic structures, and their high-Q character is usually compromised when the device is made small. Reducing the lateral size increases edge leakage, broadens the momentum distribution and weakens the infinite-lattice protection that makes BICs attractive in the first place. Early BIC lasing already showed that lasing could persist in finite arrays, even down to a few periods^6^. However, the broader mini-BIC problem is more subtle: how can one retain BIC-like vertical confinement while also providing in-plane confinement in a genuinely small footprint?

Several works addressed this by combining BIC physics with additional lateral confinement. Chen et al. demonstrated miniaturized BIC cavities with ultrahigh quality factors by combining lateral photonic-bandgap confinement with vertical BIC protection, reaching very high Q in small modal volumes^7^. Ren et al. then used mini-BIC cavities with quantum-dot gain to demonstrate low-threshold single-mode nanolasers^8^. Zhong et al. further advanced this direction with continuous-wave quantum-dot mini-BIC lasers^9^. Han et al. achieved confinement through topological band inversion^10^. These works established that mini-BICs can overcome the apparent incompatibility between BIC physics and small mode volume, but they often rely on a designed cavity region embedded in a larger photonic environment.

Peng et al. attack the problem from a different angle. In their 5 × 5-period device, the entire patterned photonic-crystal region is only about 15 μm². At this scale, one cannot simply assume that the structure behaves as a truncated version of an infinite BIC lattice. Edge leakage becomes a dominant part of the cavity physics. The authors therefore engineer the boundary holes, reducing their diameter relative to the central holes, to suppress leakage and recover BIC-derived lasing in an extremely small patterned region. The significance is not merely that a small cavity tends to have fewer modes. Indeed, reducing the area generally increases mode spacing and can make single-mode operation easier in a trivial spectral sense. The challenge is different: a very small BIC laser may lose the high-Q advantage that made the BIC useful in the first place. Peng et al. show that merging-BIC physics, finite-size quantization and edge engineering can be combined so that compactness does not simply destroy BIC protection.

Recently, the merging of accidental BICs with net-zero topological charge has shown to reshape the polarization texture, stretching the range of circularly polarized emission over a sizeable momentum-space domain^11^. Side by side with Peng et al., this suggests that BIC engineering may eventually move beyond selecting a single frequency and spatial mode, toward selecting a single handedness or spin channel, offering a possible conceptual route toward chiral or spin-selective BIC lasers.

The broader implication is that BIC lasers are entering a regime where topology, finite-size physics and gain competition must be designed together. Passive BIC photonics asks how radiation can be canceled. Active BIC photonics asks a harder question: after radiation is canceled for one state, which other states are also helped, and will any of them lase? Peng et al. answer by identifying a pre-merging sweet spot. The best laser is not located at the point of maximum radiative protection, but at the point where radiative protection is most selective. That principle may become a useful design rule for compact, stable and eventually electrically pumped BIC lasers.
